# Prostaglandin E_2_ stimulates Fas ligand expression via the EP1 receptor in colon cancer cells

**DOI:** 10.1038/sj.bjc.6604490

**Published:** 2008-07-22

**Authors:** G O'Callaghan, J Kelly, F Shanahan, A Houston

**Affiliations:** 1 Department of Medicine, University College Cork, National University of Ireland, Clinical Science Building, Cork University Hospital, Wilton, Cork, Ireland; 2 Alimentary Pharmabiotic Centre, University College Cork, National University of Ireland, Clinical Science Building, Cork University Hospital, Wilton, Cork, Ireland

**Keywords:** Fas ligand, prostaglandin E_2_, regulation, tumour immune evasion

## Abstract

Fas ligand (FasL/CD95L) is a member of the tumour necrosis factor superfamily that triggers apoptosis following crosslinking of the Fas receptor. Despite studies strongly implicating tumour-expressed FasL as a major inhibitor of the anti-tumour immune response, little is known about the mechanisms that regulate FasL expression in tumours. In this study, we show that the cyclooxygenase (COX) signalling pathway, and in particular prostaglandin E_2_ (PGE_2_), plays a role in the upregulation of FasL expression in colon cancer. Suppression of either COX-2 or COX-1 by RNA interference in HCA-7 and HT29 colon tumour cells reduced FasL expression at both the mRNA and protein level. Conversely, stimulation with PGE_2_ increased FasL expression and these cells showed increased cytotoxicity against Fas-sensitive Jurkat T cells. Prostaglandin E_2_-induced FasL expression was mediated by signalling via the EP1 receptor. Moreover, immunohistochemical analysis using serial sections of human colon adenocarcinomas revealed a strong positive correlation between COX-2 and FasL (*r*=0.722; *P*<0.0001) expression, and between EP1 receptor and FasL (*r*=0.740; *P*<0.0001) expression, in the tumour cells. Thus, these findings indicate that PGE_2_ positively regulates FasL expression in colon tumour cells, adding another pro-neoplastic activity to PGE_2_.

Colorectal cancer is a formidable health-care problem, being one of the leading causes of cancer death worldwide. Despite evidence that the host immune system can initiate an immune response against colon tumours, a large number of tumours continue to grow and evade immune-mediated elimination owing to the development of multiple immune escape mechanisms (Waldner *et al*, 2006). Upregulation of Fas ligand (FasL/CD95L) expression by tumour cells may represent one such mechanism (O’Connell *et al*, 1996). FasL is a member of the tumour necrosis factor (TNF) superfamily that can trigger apoptotic cell death following ligation to its receptor Fas (CD95/APO-1) on sensitive cells (Nagata and Golstein, 1995). Many human cancers, including colon cancer, express FasL, with growing evidence supporting a role for FasL in colon carcinogenesis (Houston and O’Connell, 2004; Whiteside, 2007). Upregulation of FasL expression is an early event in colon carcinogenesis (Bennett *et al*, 2001; Belluco *et al*, 2002), with tumour-expressed FasL being associated with apoptosis and loss of tumour-infiltrating lymphocytes (TIL) *in vivo*, and triggering the death of Fas-bearing sensitive cells *in vitro* (O’Connell *et al*, 1996; Okada *et al*, 2000). Engagement of Fas by FasL can also trigger proliferation of tumour cells (Lambert *et al*, 2003; Li *et al*, 2008) and increase tumour cell motility and invasiveness (Barnhart *et al*, 2004). Moreover, downregulation of FasL expression in colon tumour cells was recently shown to result in reduced tumour development and growth in immune-competent mice *in vivo* (Ryan *et al*, 2005). However, despite this, little is known about the molecular mechanisms underlying FasL expression in tumours.

Numerous studies have shown that cyclooxygenase-2 (COX-2) plays an important role in colon carcinogenesis (Cao and Prescott, 2002). Cyclooxygenase is a rate-limiting enzyme in the conversion of arachidonic acid to prostaglandins. Two isoforms of COX have been characterised, COX-1 and COX-2 (Williams and DuBois, 1996). Cyclooxygenase-1 is constitutively expressed, generating prostaglandins for normal physiological processes. In contrast, COX-2 is usually undetectable in most normal tissues, but can be induced rapidly by a variety of agents, including LPS, cytokines, growth factors and tumour promoters. Overproduction of COX-2 and its major metabolite prostaglandin E_2_ (PGE_2_) has been found in many human cancers, including colorectal tumours (Rigas *et al*, 1993; Eberhart *et al*, 1994; Sano *et al*, 1995), with COX-2 levels increased in about 90% of colon cancers and ∼50% of pre-malignant colorectal adenomas (Eberhart *et al*, 1994).

As increased expression of COX-2 and FasL in colon tumours has been widely observed, we tested the hypothesis that the COX signalling pathway may play a role in FasL expression in colon tumours. Here we provide evidence that the COX signalling pathway, and in particular PGE_2_, increases FasL expression in colon tumour cells. We also show that PGE_2_-induced FasL expression is mediated by the EP1 receptor. Consistent with these findings, we observed a strong positive correlation between COX-2 expression and FasL, and between EP1 receptor expression and FasL in human colon tumour cells *in vivo*. Collectively, these results support a role for PGE_2_ as a critical mediator of FasL expression in colon tumours.

## Materials and Methods

### Reagents

Prostaglandin E_2_, SC19220 and arachidonic acid were obtained from Cayman Chemical Company (Ann Arbor, MI, USA). SC-560 and SC-791 were purchased from Calbiochem (San Diego, CA, USA). rhFas:Fc was obtained from Alexis Corp. (Lausen, Switzerland).

### Cell lines and culture conditions

HT29 human colon adenocarcinoma cells and Jurkat T cells were obtained from the ATCC (Rockville, MD, USA). HCA-7 cells were a generous gift from Susan Kirkland (University of London). WIDR, SW480 and Caco-2 cells were obtained from Ken Nally (University College Cork). HT29, WIDR, SW480, Caco-2 and HCA-7 cells were maintained in DMEM containing 10% fetal calf serum (FCS) and penicillin–streptomycin, whereas Jurkat T cells were cultured in RPMI-1640 supplemented with 10% FCS and penicillin–streptomycin.

### RNA interference

The targeted sequences that effectively mediate the silencing of COX-1 (si*GENOME SMART*pool, M-004556-00), COX-2 (si*GENOME SMART*pool, M-004557-01), EP1 (ON-TARGET*plus SMART*pool, L-005711-00), EP2 (ON-TARGET*plus SMART*pool, L-005712-00) and EP4 (ON-TARGET*plus SMART*pool, L-005714-00) expression were obtained from Dharmacon Research (Lafayette, CO, USA). HCA-7 and HT29 cells were seeded 24 h before transfection at a concentration achieving 70% confluence at the time of transfection. The transfection was carried out in serum-free medium using Dharmafect 4 transfection reagent (Dharmacon Research). Cells were transfected with 100 nM of COX-1 small interfering RNA (siRNA), COX-2 siRNA, EP1 siRNA, EP2 siRNA, EP4 siRNA or irrelevant control siRNA according to the manufacturer's protocol. At 48 h after transfection, cells were treated with PGE_2_ for 24 h. Depletion of protein was confirmed by real-time RT-PCR and immunoblotting. Culture medium was collected and stored at −20°C for subsequent measurement of PGE_2_ levels by ELISA. Levels of FasL in cell lysates were determined by real-time RT-PCR and immunoblotting.

### Western blot analysis

Cells were seeded at 5 × 10^5^ cells per well in six-well plates and serum-deprived for 48 h before treatment for 24 h with either serum-free medium containing vehicle or PGE_2_, unless otherwise stated. For inhibitor studies, cells were pretreated for 1 h with the inhibitor, before the addition of PGE_2_. Cells were washed in PBS and lysed on ice in 50 mM Tris-HCl, pH 8, containing 150 mM NaCl and 1% Triton X-100, supplemented with the complete-TM mixture of protease inhibitors (Roche Molecular Biochemicals, Indianapolis, IN, USA). Protein concentrations were measured using the BCA Protein assay (Pierce, Rockford, IL, USA). Proteins (40 *μ*g protein per lane) were separated on a 10% SDS–polyacrylamide gel, transferred to a nitrocellulose membrane and then probed with one of the following primary antibodies: anti-FasL (N-20/1 : 200 dilution), anti-Fas (C-20/1 : 1000), anti-COX-1 (C-20/1 : 250) (Santa Cruz Biotechnology, Santa Cruz, CA, USA), anti-FasL (Ab-1/1 : 100 dilution) (Calbiochem), anti-TNF-related apoptosis-inducing ligand (anti-TRAIL; 1 : 500 dilution), anti-COX-2 (1 : 1000), anti-EP1 (1 : 300), anti-EP2 (1 : 500), anti-EP4 (1 : 500) (Cayman Chemical Company). As an internal control, all membranes were subsequently stripped of the first antibody and reprobed with anti-*β*-actin-specific antibody (AC-74; 1 : 10 000 dilution; Sigma, St Louis, MO, USA). The antigen–antibody complex was detected by incubating the membranes for 1 h at room temperature with the appropriate horseradish peroxidase (HRP)-conjugated secondary antibody (Dako Corp., Carpinteria, CA, USA). Peroxidase activity was detected with the enhanced chemiluminescence system (Pierce) and analysed using Scion Image analysis software (Scion Inc., Frederick, MD, USA). Changes in protein expression were determined after normalising the band intensity of each lane to that of *β*-actin.

### Quantification of mRNA

Total RNA was isolated from cultured cells using the Agilent Total RNA isolation kit (Agilent Technologies, Santa Clara, CA, USA) according to the manufacturer's instructions and cDNA synthesised using AMV reverse transcriptase, random hexanucleotide primers, RNasin (40 U) and dNTPs (500 *μ*M) (Promega, Madison, WI, USA). Quantitative PCR was then performed in triplicate using an ABI PRISM 7500 Sequence Detection System and commercially available TaqMan probes (Applied Biosystems, Foster City, CA, USA). Briefly, cDNA in 22.5 *μ*l of water was mixed with 2.5 *μ*l of 20 × TaqMan Gene Expression assay primer and probe mix (*FasL*=Hs00181225, *TRAIL*=Hs00366272, *EP1*=Hs00168752, *EP2*=Hs00168754, *EP3*=Hs00168755, *EP4*=Hs00168761, glyceraldehyde-3-phosphate dehydrogenase (*GAPDH*)=4326317E) and 25 *μ*l of 2 × TaqMan Gene Expression master mix in a 96-well optical reaction plate. The following PCR conditions were used: 50°C for 2 min, then 95°C for 10 min, followed by 50 cycles at 95°C for 15 s and 60°C for 1 min. The expression level of the target genes was normalised to internal GAPDH. Data were analysed using Microsoft Excel and calculated using the relative standard curve method (ABI, User Bulletin 2).

### Prostaglandin E_2_ assay

Prostaglandin E_2_ production was measured by ELISA, according to the manufacturer's instructions (R&D Systems, Minneapolis, MN, USA).

### Co-culture experiment

HCA-7 and HT29 cells were plated in triplicate in 96-well plates and allowed to reach ∼70% confluency. Cells were serum-starved for 48 h, followed by treatment with serum-free medium containing vehicle (0.1% DMSO), 0.5 or 1.0 *μ*M PGE_2_ for 24 h. Cells were then fixed with 1.5% paraformaldehyde and washed with PBS. Jurkat cells were added at a concentration of 5 × 10^4^ cells per well. To block FasL-mediated cytotoxicity, Jurkat cells were co-cultured with paraformaldehyde-fixed 1.0 *μ*M PGE_2_-treated cells in the presence of recombinant Fas:Fc (10 *μ*g ml^−1^). After 24 h, Jurkat cell viability was assessed by measuring the increase in fluorescence intensity associated with the cellular reduction of resazurin to resorufin, according to the manufacturer's instructions (R&D Systems). Briefly, the cells were pulsed with 44 *μ*M resazurin solution and 1 h later fluorescence was measured at 535/590 nm using a standard spectrophotometer in bottom reading mode. This fluorescence was expressed as a percentage of that shown by control Jurkat T cells in RPMI-1640 only, after subtraction of background fluorescence.

### Immunofluorescent detection of FasL

Cells were seeded at 2 × 10^5^ cells per well in six-well plates containing sterile coverslips. After serum-starving for 48 h, cells were treated with serum-free medium containing 0.5, 1.0 or 2 *μ*M PGE_2_ for 24 h. Cells were fixed with 1.5% paraformaldehyde, permeabilised with 0.2% Triton X-100 in PBS and nonspecific binding of antibody was blocked with 10% normal goat serum for 30 min at room temperature. Cells were incubated overnight at 4°C with anti-FasL-specific antibody (G247-4; Pharmingen, San Diego, CA, USA) diluted 1 : 50. Antibody binding was localised using an FITC-labelled secondary antibody (Dako Corp.). Coverslips were mounted on slides with anti-fading mounting media (Dako Corp.) and visualised using a fluorescent microscope when dry.

### Human colon adenocarcinoma tissues and immunohistochemistry

Formalin-fixed, paraffin-embedded human colonic adenocarcinomas (*n*=27) were obtained from the archives of the Mercy Hospital, Cork, following a protocol approved by the Cork Teaching Hospitals Clinical Research Ethics Committee. None of the cases used in this study had patient identifiers and strict confidentiality was maintained. None of the patients had received chemotherapy, radiotherapy or immunotherapy before tissue collection.

Tissue sections were de-paraffinised in xylene and rehydrated before analysis. Antigen retrieval was performed by microwave irradiation in 0.01 M citrate buffer, pH 6.0. Slides were washed and endogenous peroxidase was quenched with 3.0% hydrogen peroxide in methanol for 5 min. Nonspecific binding was blocked with 5% normal serum in wash buffer for 1 h. Serial sections were incubated overnight at 4°C with monoclonal FasL (G247-4) antibody (Pharmingen) at 5 *μ*g ml^−1^, rabbit polyclonal COX-2 antibody (Santa Cruz Biotechnology) at 0.5 *μ*g ml^−1^, rabbit polyclonal EP1 antibody (Cayman Chemical Company) at 0.27 *μ*g ml^−1^ or goat polyclonal COX-1 antibody (Santa Cruz Biotechnology) at 0.133 *μ*g ml^−1^. Antibody binding was localised using a biotinylated secondary antibody, avidin-conjugated HRP and DAB substrate, contained within the Vectastain ABC detection kit (Vector Laboratories, Burlingame, CA, USA). Slides were counterstained with haematoxylin and mounted. Parallel negative controls were performed for each antibody, using rabbit (COX-2, EP1) IgG, mouse (FasL) IgG or normal goat serum (COX-1) instead of primary antibody.

Neoplasms displaying immunoreactivity in more than 5% of the tumour cells were regarded as positive. Semiquantitative analysis of the immunohistochemical score for COX-2, COX-1, EP1 and FasL staining was performed by light microscopy for each tumour using a scoring system that included the extent (0=negative; 0.25=1–25% of cells stained; 0.5=26–50% of cells stained; 0.75=51–75%; 1=>75% of cells stained) and intensity (0, negative; 1, weak; 2, moderate; 3, strong; 4, intense) of staining. The scores from the two scales were multiplied by each other to give a single immunohistochemical score. For each tumour, 10 random high-power fields were scored.

### Statistics

All experiments were performed in triplicate unless otherwise indicated, and mean values were presented as mean±s.e.m. Paired Student's *t*-test was performed to compare the data between indicated groups. Spearman's rank correlation test was used for correlation between the expression of FasL and COX-1, COX-2 and EP1 in colon tumour tissue. A value of *P*<0.05 was considered statistically significant.

## Results

### Prostaglandin E_2_ induces FasL mRNA and protein expression in colon cancer cells

**Figure 1:**
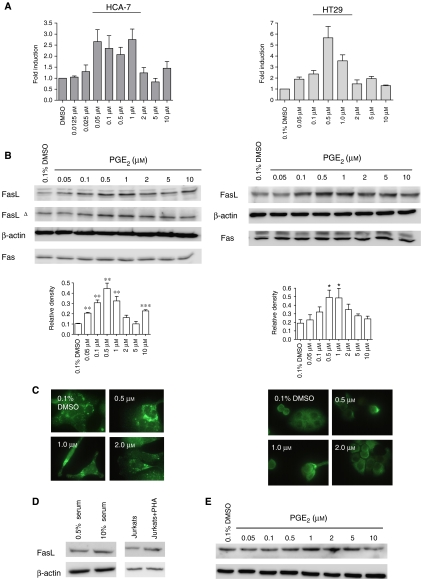
Prostaglandin E_2_ induces FasL expression in colon cancer cells. (**A**) HCA-7 cells and HT29 cells were cultured in serum-free medium for 48 h before treatment with PGE_2_. After 24 h, total RNA was extracted and was reverse transcribed to cDNA. FasL mRNA levels were measured by quantitative real-time RT-PCR. Columns indicate fold induction relative to control (DMSO). Mean±s.e.m. values are plotted. (**B**) Following the isolation of total cellular protein from cells treated as in (**A**), equal amounts of protein were separated by SDS–PAGE and visualised with anti-FasL (N-20), anti-FasL^Δ^ (Ab-1), anti-Fas or anti-*β*-actin. Semiquantitative analysis of FasL expression was determined by calculating the ratio between FasL protein and *β*-actin protein from three independent experiments. Mean±s.e.m. values are plotted (^*^P<0.05, ^**^P<0.01; compared with DMSO control; Student's *t*-test). (**C**) Cells were treated as in (**A**) and FasL protein was detected by immunofluorescence. (**D**) HCA-7 cells were serum-starved for 48 h and thereafter the medium was replaced with complete medium (10% serum) for 24 h. As a control for FasL detection, Jurkat T cells were treated with 2.5 *μ*g ml^−1^ PHA for 24 h, which has been shown to increase FasL expression in these cells. Cellular lysates were analysed by Western blotting using antibodies against FasL and *β*-actin. (**E**) HCA-7 cells were cultured in complete medium followed by treatment with PGE_2_ for 24 h. Cellular isolates were treated as in (**D**).

Numerous studies have shown that PGE_2_ produced via the COX-2 signalling pathway plays a crucial role in the development of colon cancer (Kamei *et al*, 2003). To determine if PGE_2_ induces FasL expression in colon cancer cells, we analysed the effect of PGE_2_ on FasL expression in HCA-7 human colon cancer cells. HCA-7 cells have high endogenous levels of COX-2, PGE_2_ and FasL. As COX-2 is an inducible gene that is regulated by, among other factors, serum (Dubois *et al*, 1998), HCA-7 cells were serum-starved before treatment with increasing concentrations of PGE_2_, ranging from low (0.025 *μ*M) to high (10 *μ*M), and FasL expression was analysed by real-time RT-PCR, Western blotting and immunofluorescence. As shown in Figure 1, PGE_2_ increased FasL expression at both the mRNA and protein level, with maximum induction of FasL mRNA occurring at 1.0 *μ*M PGE_2_ (>2.7-fold induction) (Figure 1A). Analysis of protein production by Western blotting and immunofluorescence showed that FasL protein also peaked at relatively low levels of PGE_2_ (0.5–1.0 *μ*M) (Figure 1B and C), whereas the levels of the Fas receptor were unaffected. This increase in FasL protein was confirmed using two different anti-FasL-specific antibodies. Prostaglandin E_2_ engagement also increased FasL expression in HT29 colon cancer cells. Relative to HCA-7 cells, HT29 cells have low levels of COX-2 and PGE_2_. However, there was an ∼5.7- and 3.6-fold induction in FasL mRNA following incubation with 0.5 and 1.0 *μ*M PGE_2_, respectively (Figure 1A), with FasL protein also peaking at 0.5–1.0 *μ*M PGE_2_ (Figure 1B and C). Based on these findings, a concentration of 1.0 *μ*M PGE_2_ was used in all subsequent experiments.

Since serum deprivation induces cell cycle arrest, and FasL expression in T cells has been reported to be cell cycle sensitive, we also determined if PGE_2_ induced FasL expression in cells grown in complete medium (10% serum). The addition of serum to serum-starved cells increased FasL expression in these cells by ∼40% as assessed by Western blotting (Figure 1D). However, the addition of serum also induced an increase in PGE_2_ secretion by these cells (data not shown). Thus, we investigated if PGE_2_ induces FasL expression in cells grown in complete medium. The addition of PGE_2_ increased FasL expression in HCA-7 cells and this increase was ∼2.5 times (1 *μ*M PGE_2_) greater than that induced by serum alone (Figure 1E), suggesting that PGE_2_ induces FasL expression independently of entry into the cell cycle. Interestingly, this induction of FasL by PGE_2_ also exhibited a ‘bell-shaped’ dose–response relationship, similar to that obtained following PGE_2_ treatment of serum-starved cells. This ‘bell-shaped’ dose–response relationship has been previously demonstrated in SW1116 cells on measuring proliferation in response to PGE_2_ and was suggested to be due to differential activation of the EP receptors at different concentrations of PGE_2_ (Qiao *et al*, 1995). Prostaglandin E_2_ binds to and activates four different receptor subtypes, EP1, EP2, EP3 and EP4 (Bos *et al*, 2004), suggesting that one (or more) EP receptor is involved in inducing FasL expression in colon cancer cells.

**Figure 2:**
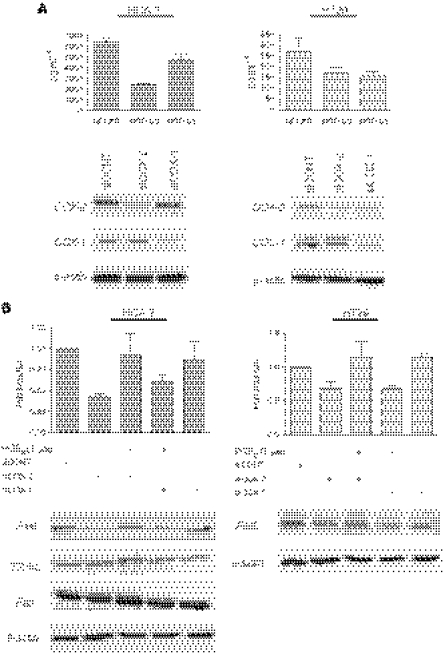
Suppression of either COX-1 or COX-2 by RNAi reduces FasL expression in HT29 and HCA-7 colon cancer cells. (**A**) HCA-7 and HT29 cells were transfected with control nonspecific RNAi (siCONT), COX-2-specific RNAi (siCOX-2) or COX-1-specific RNAi (siCOX-1). After 48 h, cell culture medium and cellular lysates were collected. Levels of PGE_2_ in the cell culture medium were determined by ELISA. Mean±s.e.m. values are plotted. Changes in COX-1 and COX-2 protein levels were detected by Western blotting. (**B**) RNA was extracted from cells treated as in (**A**) and reverse transcribed to cDNA. FasL mRNA levels were measured by quantitative real-time RT-PCR. Columns indicate fold induction relative to siCONT-treated cells. Mean±s.e.m. values are plotted. Cellular lysates were analysed by Western blotting using antibodies raised against FasL, TRAIL, Fas or *β*-actin.

**Figure 3:**
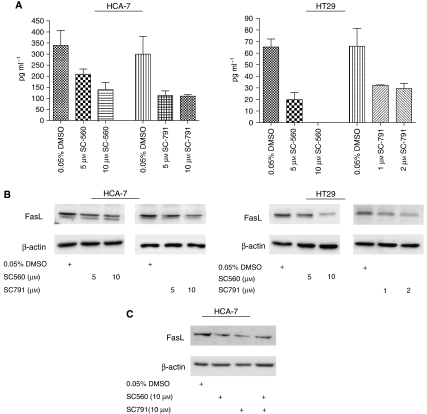
Inhibition of COX-1 using SC-560 or that of COX-2 using SC-791 reduces FasL expression in colon cancer cells. (**A**) HCA-7 and HT29 cells were serum-deprived for 48 h before treatment with SC-560 or SC-791. After 1 h, cell culture medium was collected and the levels of PGE_2_ were determined by ELISA. Mean±s.e.m. values are plotted. (**B**) HCA-7 and HT29 cells were serum-deprived for 48 h, treated with SC-560 or SC-791 and 24 h later cell lysates were collected. Lysates were analysed by Western blotting using antibodies raised against FasL and *β*-actin. Data shown are representative of three independent experiments. (**C**) HCA-7 cells were serum-deprived for 48 h, treated with 10 *μ*M SC-560 and/or 10 *μ*M SC-791 and 24 h later cell lysates were collected and treated as in (**B**).

Furthermore, depletion of COX-1 and COX-2 by RNA interference (RNAi) reduced FasL expression in both HCA-7 and HT29 cells. Although COX-2 is the isoform that is overexpressed in colon cancer, evidence from mouse experiments has also implicated COX-1 as a causal agent in COX-induced colorectal carcinogenesis (Chulada *et al*, 2000), with production of PGE_2_ being the common denominator. Thus, the expression of both COX-1 and COX-2 was reduced by RNAi, and the efficiency of depletion was verified by PGE_2_ ELISA and Western blotting (Figure 2A). Suppression of COX-2 and COX-1 by RNAi reduced FasL expression in both HCA-7 and HT29 cells, as evidenced by a reduction in FasL mRNA and protein production (Figure 2B). Cyclooxygenase-2 depletion resulted in a >55% reduction in FasL mRNA in HCA-7 cells and a >30% reduction in FasL mRNA in HT29 cells, as assessed by real-time RT-PCR, whereas FasL mRNA was reduced by >38 and >32% in HCA-7 and HT29 cells, respectively, by COX-1 RNAi. Western blot analysis confirmed these findings at the protein level, with siCOX-2 and siCOX-1 significantly reducing FasL protein in HCA-7 (*P*<0.01 and *P*<0.01, respectively) and HT29 (*P*<0.01 and *P*<0.05, respectively) cells, as assessed by semiquantitative densitometric analysis. This effect was specific for FasL and did not affect TRAIL, another death-inducing member of the TNF superfamily, or Fas receptor, as assessed by real-time RT-PCR (data not shown) and Western blotting (Figure 2B). Importantly, PGE_2_ restored FasL production in both cell lines in the presence of COX-2 and COX-1 RNAi, demonstrating that COX-derived PGE_2_ is an important mediator of FasL expression in colon cancer cells. These findings were also confirmed using COX-1- and COX-2-specific inhibitors. As shown in Figure 3, treatment with SC-560 (COX-1-specific inhibitor) or SC-791 (COX-2-specific inhibitor) reduced PGE_2_ secretion into the culture medium in both systems, with a concomitant reduction in FasL protein. Interestingly, inhibition of both COX-1 and COX-2 did not completely abrogate FasL expression (Figure 3C), suggesting that in colon tumour cells PGE_2_ is only one of the factors responsible for regulating FasL expression.

**Figure 4:**
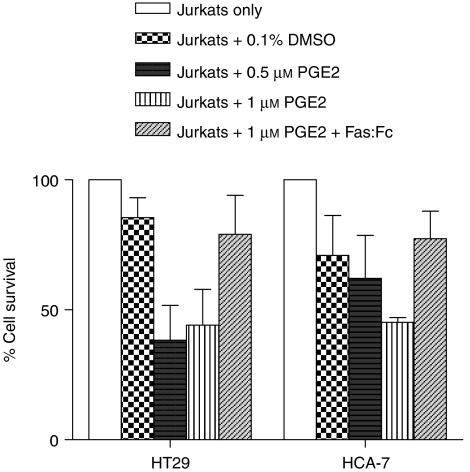
Reduction in Jurkat T-cell viability following co-culture with colon cancer cells treated with PGE_2_. HT29 and HCA-7 cells were serum-deprived for 48 h, followed by treatment for 24 h with 0.1% DMSO, 0.5 or 1.0 *μ*M PGE_2_. Cells were fixed and then co-cultured with Jurkat T cells for 24 h. Jurkat T-cell viability was determined by resazurin reduction. To block FasL-mediated apoptosis, Jurkat T cells were also co-cultured with 1.0 *μ*M PGE_2_-treated colon cancer cells in the presence of 10 *μ*g ml^−1^ recombinant Fas:Fc blocking protein. Data denote mean±s.e.m. and are representative of two independent experiments. Viability is expressed as an index (% of control Jurkat T cells maintained in medium only).

To corroborate the role of PGE_2_ in inducing FasL expression at a functional level, HCA-7 and HT29 cells were treated with PGE_2_ for 24 h, washed with PBS, fixed and then co-cultured with Jurkat T cells. The Jurkat T-cell line is a Fas-sensitive cell line of T-cell origin that is widely used experimentally as a model for activated T cells. There was a 14.5 and 29.1% reduction in Jurkat T-cell viability following co-culture with control HT29 and HCA-7 cells, respectively. However, upregulation of FasL expression by PGE_2_ on HCA-7 and HT29 cells substantially increased their effector function against the Jurkat T cells (Figure 4). Following co-culture with 1.0 *μ*M PGE_2_-treated HT29 and HCA-7 colon cancer cells, Jurkat T-cell viability decreased by 55.9 and 54.8%, respectively, relative to cells cultured in medium only. This reduction in cell viability following co-culture with 1.0 *μ*M PGE_2_-treated cells was confirmed to be Fas-mediated, as it could be blocked by the addition of neutralising anti-FasL antibody.

### Prostaglandin E_2_-induced FasL expression is EP1 receptor-dependent

**Figure 5:**
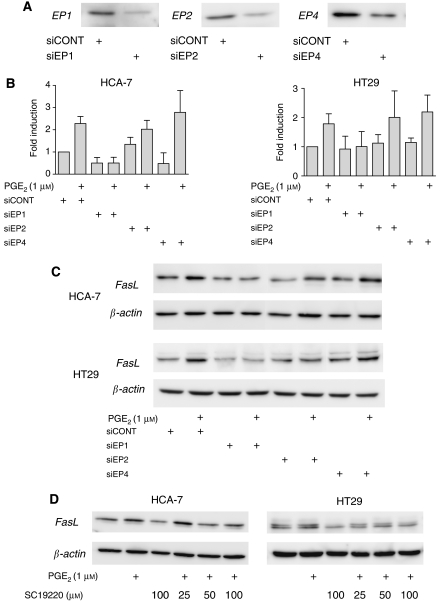
Suppression of EP1 blocks PGE_2_-induced FasL upregulation in HCA-7 and HT29 cells. (**A**) HCA-7 cells were transfected with control nonspecific RNAi (siCONT), EP1-specific RNAi (siEP1), EP2-specific RNAi (siEP2) or EP4-specific RNAi (siEP4). After 48 h, cells were lysed and successful depletion of EP1, EP2 and EP4 receptors in cells transfected with the corresponding siRNA was confirmed by Western blotting. (**B**) At 48 h after transfection with siCONT, siEP1, siEP2 or siEP4, HCA-7 and HT29 cells were treated with 1.0 *μ*M PGE_2_ for 24 h and RNA was extracted and reverse transcribed to cDNA. FasL mRNA levels were measured by quantitative real-time RT-PCR. Columns denote fold induction relative to siCONT-treated cells. Mean±s.e.m. values are plotted. (**C**) Total protein was isolated from cells treated as in (**B**) and lysates were analysed by Western blotting using antibodies raised against FasL and *β*-actin. (**D**) Cells were serum-starved for 48 h, pretreated with SC19220 for 1 h, followed by treatment with 1.0 *μ*M PGE_2_ for 24 h. Cells were lysed and analysed by Western blotting using antibodies raised against FasL and *β*-actin.

Prostaglandin E_2_ exerts its biological activity through four different receptor subtypes, EP1, EP2, EP3 and EP4 (Bos *et al*, 2004), with HCA-7 cells expressing all four receptors, as determined by real-time RT–PCR. In contrast, HT29 cells expressed only EP1 and EP4. As EP1, EP2 and EP4 have previously been reported to play a role in colon carcinogenesis, we investigated whether these receptors are involved in PGE_2_-induced FasL expression. In the first experiment, we utilised siRNA to downregulate EP1, EP2 and EP4 in HCA-7 cells. Transfection with EP1-, EP2- and EP4-specific siRNA resulted in efficient impairment of EP1, EP2 and EP4 transcription (64, 84 and 59% reduction in mRNA, respectively) and protein translation (Figure 5A). Cells were then treated with PGE_2_ and FasL production was analysed by real-time RT-PCR and immunoblotting. As shown in Figure 5B and C, suppression of EP1 prevented PGE_2_-induced FasL expression in HCA-7 cells at both the mRNA and protein levels. In contrast, EP2- and EP4-specific siRNA had little or no effect. Similar results were obtained with HT29 cells, where transfection with EP1-specific siRNA resulted in a decreased induction of FasL following treatment with PGE_2_ (Figure 5B and C).

As an alternative approach to inhibiting EP1 receptor activity, in a second experiment cells were pretreated for 1 h with SC19220 (25–100 *μ*M), which is an EP1 receptor antagonist, before the addition of PGE_2_ and FasL expression was analysed by immunoblotting. As shown in Figure 5D, SC19220 prevented the PGE_2_-induced increase in FasL expression in a dose-dependent manner, consistent with a role for EP1 receptor in mediating this effect.

**Figure 6:**
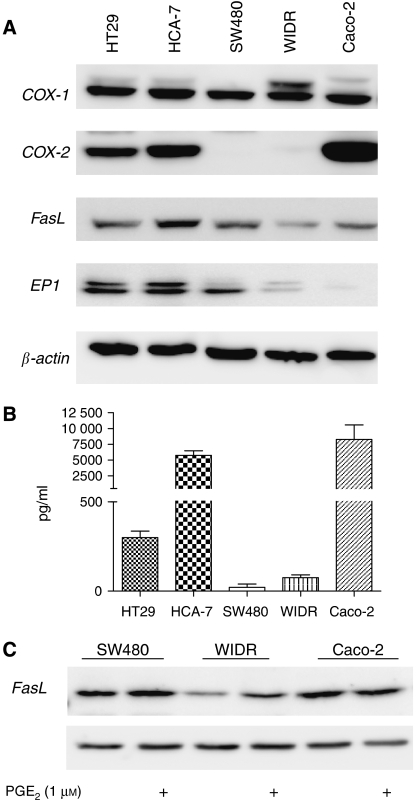
FasL expression is correlated with EP1 receptor expression in colon cancer cells *in vitro*. (**A**) Cells were seeded at 1 × 10^5^ cells per ml and after 24 h the medium was replaced with fresh growth medium. After 24 h, cell culture medium and cellular lysates were collected. Cellular lysates were analysed by Western blotting using antibodies raised against COX-1, COX-2, FasL, EP1 receptor or *β*-actin. (**B**) Levels of PGE_2_ in the cell culture medium were determined by ELISA. Mean±s.e.m. values are plotted. (**C**) SW480, WIDR and Caco-2 cells were serum-starved for 48 h before treatment with 1.0 *μ*M PGE_2_ for 24 h. Cellular isolates were separated by SDS–PAGE and visualised with anti-FasL or anti-*β*-actin.

To determine if there was any correlation between the basal expression level of FasL and the EP1 receptor, five different colon cancer cell lines were grown in complete media and the expression of COX-1, COX-2, FasL and EP1 was assessed by Western blotting (Figure 6A), whereas PGE_2_ secretion was determined by ELISA (Figure 6B). Four of the cell lines (HT29, HCA-7, WIDR and Caco-2) expressed all five proteins, although at very different levels. (COX-2 was detectable in WIDR cells on increased exposure). In contrast, SW480 cells were negative for COX-2, whereas Caco-2 cells expressed very low levels of EP1 receptor. Interestingly, although both HCA-7 and Caco-2 cells strongly expressed COX-2 and PGE_2_, HCA-7 cells exhibited much stronger expression of FasL than Caco-2 cells, which is likely due to the much higher expression level of EP1 receptor found in these cells. HT29 cells also strongly expressed FasL, despite producing much lower levels of PGE_2_. However, HT29 cells also strongly expressed the EP1 receptor. SW480 and WIDR in turn secreted very low amounts of PGE_2_. However, SW480 cells expressed more EP1 receptor than WIDR cells, together with stronger basal production of FasL. Finally, 1.0 *μ*M PGE_2_ induced FasL expression in both SW480 and WIDR cells, but not in Caco-2 cells, which is likely due to the very low/negligible levels of EP1 receptor expressed by Caco-2 cells.

### Correlation between COX-1, COX-2, EP1 receptor and FasL expression in human colon cancer cells

**Figure 7:**
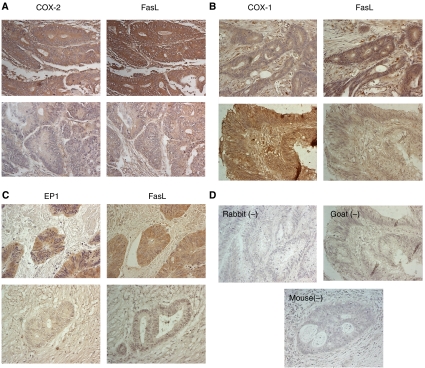
Immunohistochemical visualisation of COX-1, COX-2, EP1 receptor and FasL in human colon cancer. (**A**) Cyclooxygenase-2 and FasL, (**B**) COX-1 and FasL and (**C**) EP1 receptor and FasL immunostaining in cancer cells, with the upper and lower panels representing different colon cancer specimens. Specific protein stained brown. (**D**) Rabbit, goat and mouse controls demonstrated no staining. Representative micrographs are shown. Original magnification: × 300.

To determine if our *in vitro* data had *in vivo* relevance, COX-1, COX-2, EP1 receptor and FasL expression was analysed by immunohistochemistry on sequential sections from 27 human colon cancer tissues. Examination of stained tumour specimens revealed that 81% (22 out of 27) of the tumours expressed FasL and 96% (26 out of 27) expressed EP1, whereas COX-1 protein was detected in the neoplastic cells of 88% (22 out of 25) of tumours and COX-2 in 85% (23 out of 27) of tumours. Staining for all four proteins varied in intensity and extent both within individual tumours and between tumours, with some cells in the stromal compartments also exhibiting positive immunoreactivity. However, strong immunostaining for FasL was seen more frequently in cancer cells with strong immunostaining for COX-2 (Figure 7A) and EP1 receptor (Figure 7C). In contrast, strong immunostaining for COX-1 did not frequently coincide with strong FasL immunostaining (Figure 7B). No staining was seen when the primary antibody was substituted with isotype-matched controls (rabbit and mouse) or non-immune serum goat (Figure 7D).

To determine if expression of the proteins correlated *in vivo*, consecutive sections were scored in a blinded fashion for both the number of COX-1-, COX-2-, EP1- and FasL-positive tumour cells and the intensity of staining, and the final scores were multiplied to give a total immunohistochemical score. There was no correlation between the immunostaining scores of COX-1 and FasL. However, a strong positive correlation was observed between COX-2 and FasL (*r*=0.722; *P*<0.0001), and between EP1 and FasL (*r*=0.740; *P*<0.0001) immunostaining, indicating a possible interrelationship in the expression of these proteins.

## Discussion

Although there are conflicting reports on the role of FasL in tumorigenesis (Restifo, 2000;, O’Connell *et al*, 2001), considerable evidence now exists indicating that FasL expression by tumour cells may aid in tumour development. Studies performed *in vitro*, as well as evidence from numerous *in situ* examinations, indicate that tumour-expressed FasL is associated with apoptosis of TIL and may thus protect tumour cells from destruction by the immune system (O’Connell *et al*, 2001; Whiteside, 2007). Tumour-expressed FasL has also been shown to be associated with metastasis of tumour cells to the lymph nodes and liver (Mann *et al*, 1999; Younes *et al*, 2000), whereas ligation of Fas by FasL can stimulate cell proliferation (Lambert *et al*, 2003; Li *et al*, 2008), and increase tumour cell motility and invasiveness (Barnhart *et al*, 2004; Peter *et al*, 2007). Furthermore, recent studies suggest that tumour-expressed FasL may contribute to tumour growth in cancers associated with inflammation, in part through the induction of chemotactic factors (Matsumoto *et al*, 2007). Together, these findings suggest that the FasL signalling pathway plays a critical role in tumour development and immune evasion, and that suppression of FasL expression may aid in the treatment of certain cancers. In support of this, we have recently shown that specific inhibition of FasL expression in colon cancer cells significantly retards tumour formation in immune-competent syngeneic mice (Ryan *et al*, 2005). However, despite the role of FasL in tumorigenesis, little is known about the regulation of this molecule in tumour cells.

Increased PGE_2_ production has been shown to be strongly associated with colorectal neoplasia, increasing critical pro-survival and anti-apoptotic factors that favour cell growth (Wang and Dubois, 2006). However, the effector genes downstream of PGE_2_ have not been well characterised. In this study, we show using a variety of methods that PGE_2_ regulates FasL expression in colon cancer cells, suggesting that PGE_2_ may also play a role in FasL-mediated tumour immune evasion. Induction of FasL *in vitro* occurred at relatively low concentrations of PGE_2_ (∼0.5–1.0 *μ*M), with both COX-2 and COX-1 appearing to play a role in this process. However, in human colon cancer cells *in vivo*, COX-2 appears to play a more important role in the induction of FasL than COX-1, with expression of COX-2 and not COX-1 correlating with FasL expression. One potential explanation may be that although both isoforms may play a role in the induction of FasL expression through production of PGE_2_, *in vivo* COX-2 is the isoform that is increased in colon cancer, with a concomitant increase in PGE_2_ (Rigas *et al*, 1993; Eberhart *et al*, 1994). In contrast, COX-1 expression has been reported to remain constant under most physiological and pathological conditions (Eberhart *et al*, 1994; Sano *et al*, 1995). Although recent studies have shown that COX-1 may also play a role in carcinogenesis in some cancers, such as ovarian (Gupta *et al*, 2003), head and neck (Erovic *et al*, 2008) and colon cancer (Chulada *et al*, 2000), the common denominator in all these studies is an increase in the overall level of PGE_2_. Following upregulation during colon carcinogenesis, COX-2 may be the primary source of PGE_2_ in the tumour microenvironment, and thus may play a more important role in inducing FasL expression in human colon tumour cells *in vivo*.

Prostaglandin E_2_ mediates its effects by binding to and activating four different G-protein-coupled receptors, EP1, EP2, EP3 and EP4, with genetic deletion and pharmacologic manipulation of the EP receptors identifying different roles for EP1, EP2 and EP4 in intestinal tumorigenesis (Wang and Dubois, 2006). In the current study, we show that the EP1 signalling pathway is the predominant pathway that mediates PGE_2_-induced FasL expression. Prostaglandin E_2_-induced FasL was blocked by both EP1-specific RNAi and pharmacologic inhibition of EP1, but not by EP2- or EP4-specific RNAi. Moreover, a strong correlation was observed between EP1 and FasL expression in colon cancer cells *in vitro* and *in vivo*, indicating a possible interrelationship in the expression of these two proteins and together suggesting that these findings may have physiological relevance. The EP1 receptor has previously been shown to play an important role in colon carcinogenesis. The development of both aberrant crypt foci and colon tumours was significantly reduced in EP1-knockout mice following treatment with the colon carcinogen azoxymethane (Watanabe *et al*, 1999; Kawamori *et al*, 2005). Also recent studies characterising EP receptor expression in a variety of tumour types showed that expression of EP1 is increased in tumour cells relative to normal tissue (Shoji *et al*, 2004; Miyata *et al*, 2006; Rask *et al*, 2006) and is associated with tumour progression and metastasis in prostate cancer (Miyata *et al*, 2006). Similarly, FasL expression has been shown to be upregulated in tumour cells relative to normal tissue and is associated with tumour progression and metastasis (Mann *et al*, 1999; Osaki *et al*, 2001; Belluco *et al*, 2002). As inhibition of FasL expression in colon cancer cells significantly retards tumour formation in mice (Ryan *et al*, 2005), these findings suggest that targeting the EP1 receptor may help to prevent or treat colon cancer, in part through preventing FasL upregulation in tumour cells.

However, PGE_2_ has also been shown to downregulate FasL expression in T cells, inhibiting activation-induced cell death (Porter and Malek, 1999). In contrast, FasL expression has recently been shown to be induced in normal colonocytes by PGE_2_ (Kim *et al*, 2007). Our results clearly demonstrate that PGE_2_ induces FasL expression in colon tumour cells. Together, these findings suggest that PGE_2_ can have multiple effects, perhaps depending on the cell type and oncogenic state of the target cell. Interestingly, in agreement with our findings, the EP1 receptor also played a critical role in PGE_2_-mediated induction of FasL in normal colonocytes. Although in this model upregulation of FasL expression resulted in colonocyte apoptosis, tumour cells have previously been shown to exhibit enhanced resistance to Fas-mediated apoptosis (Oikonomou *et al*, 2007). Thus, upregulation of FasL expression in response to PGE_2_ and subsequent activation of the EP1 receptor, coupled with resistance to Fas-mediated apoptosis, may aid in tumour development. Furthermore, activation of the transcription factor NF-*κ*B was critical for FasL expression in toxin A-exposed colonocytes. Future studies will determine if EP1-mediated activation of NF-*κ*B also plays an important role in inducing FasL expression in colon tumour cells.

In summary, our findings demonstrate that PGE_2_ upregulates FasL expression in colon cancer cells through the EP1 receptor. Interruption of the PGE_2_ pathway *in vivo* has been shown to delay and/or prevent tumour progression in many systems. These anti-tumour effects have been attributed to various mechanisms, such as reduced angiogenesis and increased apoptosis. Our finding that the pro-inflammatory cytokine PGE_2_ upregulates FasL expression in colon cancer cells adds another pro-neoplastic activity to this pathway, that is, tumour immune evasion, and suggests that targeting the PGE_2_–EP1–FasL signalling pathway may aid in the development of new therapeutic strategies to both prevent and treat this malignant disease.
